# Management of soft-tissue sarcomas; treatment strategies, staging, and outcomes

**DOI:** 10.1051/sicotj/2017010

**Published:** 2017-03-10

**Authors:** Eyal M. Ramu, Matthew T. Houdek, Christian E. Isaac, Colleen I. Dickie, Peter C. Ferguson, Jay S. Wunder

**Affiliations:** 1 University of Toronto Musculoskeletal Oncology Unit, Mount Sinai Hospital Toronto M5G 1X5 Ontario Canada; 2 Department of Surgical Oncology, Princess Margaret Cancer Center, University of Toronto Toronto M5G 2C4 Ontario Canada; 3 Division of Orthopedic Surgery, Department of Surgery, University of Toronto Toronto M5G 2C4 Ontario Canada; 4 Department of Radiation Oncology, Princess Margaret Cancer Center, University of Toronto Toronto M5G 2C4 Ontario Canada

**Keywords:** Soft tissue sarcoma, Outcome, Management, Reconstruction

## Abstract

Soft-tissue sarcomas (STS) are a rare group of malignant tumors which can affect any age group. For the majority of patients who present with a localized STS, treatment involves a multidisciplinary team decision-making approach ultimately relying on surgical resection with or without adjuvant radiation for successful limb salvage. The goals of treatment are to provide the patient with a functional extremity without local tumor relapse. The purpose of this article is to review the treatment of extremity STS, with a focus on staging, treatment options, and outcomes.

## Introduction

Soft-tissue sarcomas (STS) are a diverse group of rare malignant tumors which arise from mesenchymal tissue. Approximately 11,000 new cases of STS are diagnosed each year in the United States, accounting for <1% of all cancers [1]. STS can occur over all age ranges, however the median age at diagnosis is 56–65 years, peaking in the 8th decade [2]. STS can arise anywhere in the body; however, the extremities account for 60% of cases, with the thigh being the most common site of disease [2]. STS are classified based on the mature tissue they resemble, with nearly 100 histologic subtypes in the World Health Organization (WHO) classification [3]. These subtypes vary based on molecular characteristics, clinical behavior, and response to treatment. Low-grade tumors may be locally invasive but rarely metastasize. Higher grade tumors exhibit more aggressive behavior with a more substantial risk of mortality due to the development of metastatic disease (predominantly to the lungs) [4].

## Etiology, clinical presentation, and diagnosis

The etiology of most STS remains unknown; however, there are certain environmental factors and genetic predispositions which have been associated with the development of some types of STS, including neurofibromatosis and Li-Fraumeni syndrome. The initial signs and symptoms of a STS may vary depending on the tumor site, subtype, and grade. Most commonly patients present with an enlarging painless mass, however tumor growth can cause pain via a mass effect on nearby neurovascular structures.

Certain tumors have a tendency to appear at a certain age (e.g. liposarcoma in adults and rhabdomyosarcoma in children). Likewise, certain STS are more common in specific anatomic locations: liposarcoma is more common in the lower extremity, whereas synovial sarcoma, epithelioid sarcoma, and fibrosarcoma are encountered more often in the upper extremity [4]. Rapid growth raises concern for a malignant diagnosis, while fluctuations in size can be seen in benign lesions such as ganglion cysts and vascular malformations. A small, soft, superficial, mobile mass is most likely to be benign [5]. Asking the patient to contract the muscle adjacent to the mass and assessing its subsequent mobility can help in defining the relationship of the mass to the underlying fascia. Rarely (<5%) STS metastasize to lymph nodes, but some histologic subtypes (synovial sarcoma, rhabdomyosarcoma, epithelioid sarcoma, clear cell sarcoma, and angiosarcoma) have a higher propensity for lymphatic spread and in those cases regional lymph nodes should be assessed [6, 7].

## Diagnosis of a soft-tissue sarcoma

There are three factors which need to be evaluated as part of the investigation of a patient with a STS: (1) local extension, (2) histological diagnosis, and (3) staging of metastases. Each of these pieces of information plays an important role in developing a patient-specific treatment plan [8, 9].

### Assessment of local extension

STS generally spread along tissue planes, compressing the surrounding tissues and typically do not violate anatomic barriers such as fascia or bone. It is unusual for a STS to invade bone, but when it occurs, bone invasion is associated with a significant reduction in overall survival [10]. Likewise the microscopic extent of tumor cells in the edema surrounding a STS, as seen on magnetic resonance imaging (MRI), could represent a cause of local recurrence if left untreated [11].

**Figure 1.**
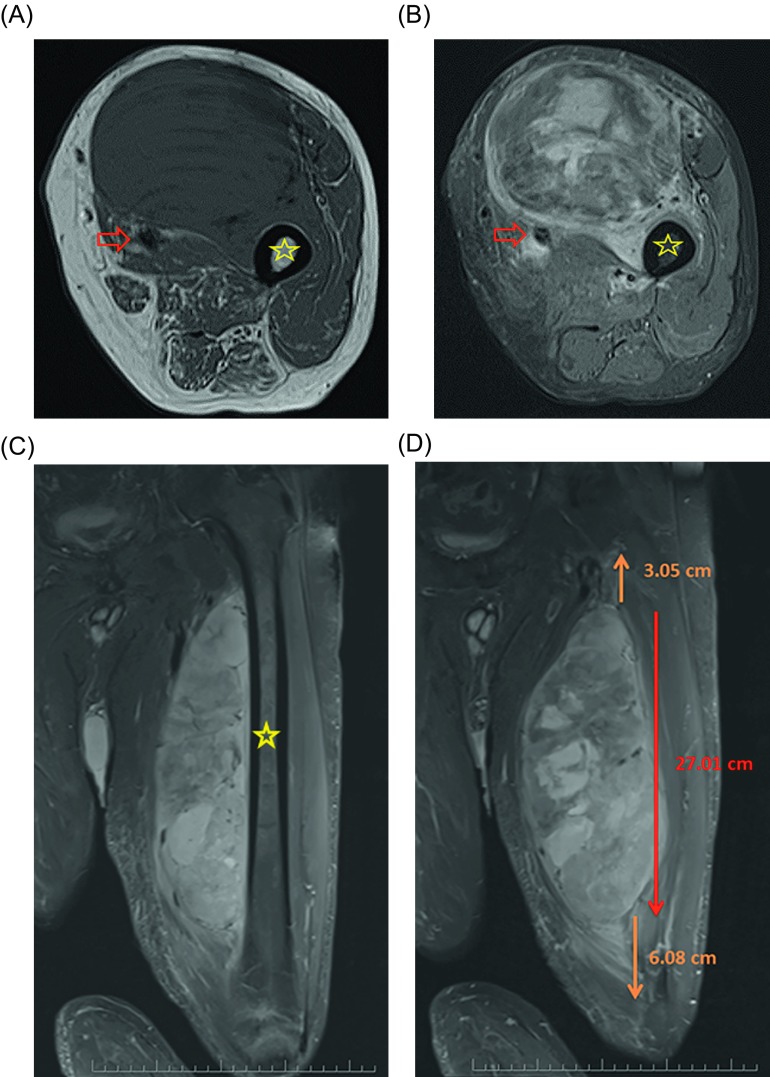
Selected T1 (A) and fat-saturated T2 (B) axial as well as fat-saturated coronal T2 (C) MRI images of a 60-year-old patient with a large, deep mass located in the anterior thigh. On the pretreatment imaging the mass was intimately associated with the femoral neurovascular bundle (arrow) as well as the periosteum of the femur (star). A biopsy was performed and showed high-grade pleomorphic rhabdomyosarcoma. The mass measured approximately 27 cm cranial/caudal however was associated with peritumoral edema which spanned nearly the entire length of the femur on coronal fat-saturated T2 (D) MRI images.

Imaging evaluation is best performed by MRI of the extremity. Plain radiographs are rarely required but can help identify bone remodeling, bone invasion, and soft-tissue calcification or ossification [12]. MRI is considered the “gold standard” for defining the local extent of the tumor and surrounding edema (Figure 1) [11]. MRI technology can reconstruct a three-dimensional model from cross-sectional images and provides pertinent anatomic information related to the tumor and its proximity to critical neurovascular structures and bone. This information is important for planning surgical excision, as the strongest predictor of local recurrence is a positive surgical margin [13, 14]. The addition of gadolinium contrast to the MRI can help differentiate between cystic areas representing hemorrhage or necrosis based on peripheral rim enhancement, and solid viable areas of tumor based on enhancement throughout the lesion. An MRI demonstrating a heterogeneous mass with predominantly low signal intensity on T1-weighted images, high signal intensity on T2-weighted images, and post-gadolinium contrast enhancement is very characteristic of a STS (Figure 1) [15]. Likewise magnetic resonance angiography is also a very useful modality in assessing the relationship of STS to adjacent neuromuscular bundle.

### Histological diagnosis

Pathological assessment is necessary to define the histologic subtype and grade and should be obtained prior to definitive treatment if there is concern for a STS. Different subtypes can vary in their clinical behavior and response to treatment and histologic grade has been identified as one of the strongest predictors of metastatic risk and disease-free survival [14, 16–19].

In the case of a suspected STS, biopsy should be performed prior to excision in order to avoid inadequate surgery. Two types of biopsies are commonly used today: needle (fine-needle aspiration (FNA), core needle biopsy (CNB)) and open biopsy (incisional, excisional). Needle biopsies are less time consuming, relatively inexpensive, cause minimal morbidity, limited soft-tissue contamination, and can be performed in an outpatient clinic setting [20–23]. FNA may be able to establish the presence of malignancy, but CNB is usually required as it provides the pathologist with an adequate tissue sample, and it has been suggested that 4–6 cores of tumor tissue are necessary for an accurate diagnosis [20–24].

Incisional biopsy provides a larger amount of tissue for histologic assessment and grading and thereby provides a better estimate of prognosis [24]. An operative biopsy should be carefully planned using a longitudinal/extensile incision that can be excised as part of a definitive surgical incision should the final diagnosis confirm a STS. Careful hemostasis is critical to minimize the risk of soft-tissue contamination by hematoma. Excisional biopsy of an extremity mass without a definitive diagnosis is usually reserved for small, superficial, and mobile masses which are most likely to be benign, or situations in which the diagnosis is in doubt but excision can be easily performed with a true wide margin.

### Staging

The diagnostic workup is completed by staging investigations for regional and distant metastases. The two most commonly used staging systems are the American Joint Committee on Cancer (AJCC) staging system and the Musculoskeletal Tumor Society (MSTS) staging system [25]. Both of these staging systems utilize the local extent of the tumor (size and depth for AJCC vs. compartment status for MSTS), histologic grade, and the presence or absence of metastases.

Extremity STS most commonly metastasize hematogenously with a strong predilection for the lungs and 10% of patients will have detectable pulmonary disease at the time of initial presentation [26]. The initial workup should include systemic staging by chest computed tomography (CT)-scan to evaluate the lungs. A bone scan can be used to evaluate for the rare occurrence of metastatic bone disease although it can be negative even in the presence of osseous metastases. In addition to a bone scan, a positron emission tomography (PET) scan can be helpful in staging of recurrent disease. If the biopsy confirms the diagnosis of a subtype of STS prone to metastasize to lymph nodes, imaging of the regional lymph nodes with a CT scan should be undertaken.

## Treatment of extremity soft-tissue sarcomas

Every patient with a soft-tissue sarcoma in the upper or lower extremity will require an individualized treatment plan. Various patient, tumor, and anatomic characteristics need to be evaluated in a multidisciplinary setting in order to generate the optimal treatment plan.

Most localized extremity STS are best treated surgically with or without radiation therapy. Chemotherapy is usually reserved for management of patients with metastatic disease either at presentation or following resection of the primary tumor, or less commonly for attempting to facilitate local tumor down-staging for very extensive lesions which might not otherwise be amenable to limb sparing surgery. Isolated lymph node metastases are somewhat of an exception however as long-term survival is still possible following surgical resection [7]. The overall treatment goal is to achieve maximal oncologic control and render the least functional impairment. As is the case in all areas of medicine, a thoughtful “risks-benefits” discussion is critical and each patient should be involved in the multidisciplinary decision-making process.

The term “oncologic control” refers to minimizing each patient’s risk of local and systemic recurrence with current treatment modalities. Historically, many soft-tissue sarcomas of the extremity were treated with amputation or radical resection alone. Although this approach provided a high degree of local tumor control, it was at the expense of residual limb function and yet still left patients at risk for developing metastatic disease. The introduction of adjuvant radiotherapy and developments in cross-sectional imaging, particularly MRI, has allowed more conservative resection margins to be considered safe, thereby extending the indications for limb salvage. In general, modern limb-salvage techniques can achieve comparable oncologic control with superior functional outcomes compared to amputation. As a result, primary amputation for management of extremity STS is rarely indicated except for situations with very extensive and locally invasive disease (Table 1) [27].

**Table 1.** Indication for primary amputation for extremity soft tissue sarcomas.

Indications for amputation [27]
1) Limb salvage would result in inadequate function of the limb.
2) Composite tissue involvement.
3) Prior unplanned excision (resulting in widespread tissue contamination) with exposed multiple neurovascular structures and/or bone.
4) Elderly patients with major medical comorbidities who are unlikely to tolerate a major operation (a potential indication for primary amputation).

Radiation therapy is recommended for all STS where surgery will provide less than a wide negative resection margin. Adjuvant radiation can be given preoperatively or postoperatively and this was the subject of a randomized clinical trial (RCT) [28]. Preoperative radiation typically prescribes a total of 50 Gy delivered in 2 Gy daily fractions over five weeks followed by surgery four to six weeks after the completion of radiation. In comparison, postoperative radiation begins approximately four to six weeks after surgery or once the wound has adequately healed, and typically involves 30–33 daily fractions delivered over six weeks to a total of 60–66 Gy. Preoperatively the radiation field encompasses the tumor and an additional surrounding region to account for tissues that may have microscopic disease. Postoperatively a larger dose of radiation is given to a larger target volume because of the theoretical issues of tissue hypoxia, and the fact that the entire surgical wound needs to be included in the treatment field. Preoperative radiation is associated with a significantly higher wound complication rate, [28] which can be partially minimized by the timing of surgery [29, 30]. Wound complications following preoperative radiation can complicate patients’ short-term outcome but are usually resolvable and have little impact on long-term function [31, 32]. In comparison, patients treated with postoperative radiation are more likely to develop significant fibrosis, lymphedema, joint stiffness, and pain which correlate with significantly worse long-term functional outcome [31, 32]. Long-term follow-up of patients in this randomized trial demonstrated the sequelae following postoperative radiation can be permanently disabling [31]. Importantly, there were no differences in local or systemic disease recurrence between patients treated with preoperative or postoperative radiation.

In the above RCT, patients who received 50 Gy preoperatively and had positive resection margins were treated with an additional 16 Gy radiation boost following surgery. Two subsequent studies showed that this postoperative radiation boost increased the total dose of radiation without offering any detectable advantage in local control so this practice has since been abandoned at our institution, as well as many others in North America, but has yet to become a widely accepted treatment policy [33, 34]. In cases with particularly radiosensitive tumors, such as myxoid liposarcoma, preoperative radiation can lead to substantial tumor shrinkage prior to surgery and is associated with excellent outcomes [35–37]. Although preoperative radiation is associated with an increased risk of early wound complications, it does not impede successful microvascular anastomosis in cases needing free flaps for soft-tissue reconstruction, but avoids direct radiation to a free tissue transfer, rotational flap, or skin graft when needed for wound coverage [38].

**Figure 2.**
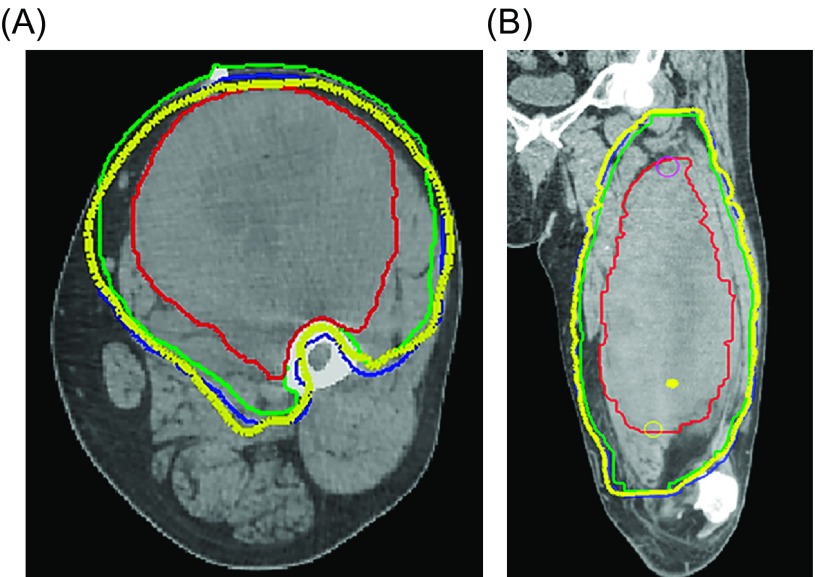
Preoperative radiotherapy planning volumes for the patient in Figure 1 are shown on axial (A) and coronal (B) CT images. The Gross Tumor Volume (GTV) is demonstrated by the solid red contour; Clinical Target Volume (CTV) is demonstrated by the green solid contour; Planning Target Volume (PTV) is shown by the blue solid contour; and the thick yellow line represents the prescribed radiotherapy dose volume. Note that intensity-modulated radiotherapy (IMRT) was used to adequately encompass the radiotherapy target volume while avoiding the bone by sculpting the high dose volume around the femoral cortex for protection purposes (A), while also accounting for the peritumoral edema surrounding the lesion (B) which was demonstrated on the coronal fat-saturated T2 post-gadolinium image in Figures 1C and 1D.

Image-guided intensity-modulated radiation therapy (IMRT) is becoming the standard of care for sarcoma patients at our institution and can be provided in either the preoperative or postoperative setting (Figure 2). The potential advantage of IMRT is its ability to “sculpt” the treatment volume and thereby provide less radiation to surrounding normal tissues, such as skin, bone, and neurovascular structures, without compromising target coverage [39]. In a Phase II clinical trial for patients with lower extremity STS, preoperative IMRT substantially decreased the radiation dose to planned overlying skin flaps as well as bone, decreased the wound complication rate and need for surgical intervention for wound complications, and led to a higher rate of primary wound closure and less need for soft-tissue flaps [29, 30, 39]. In addition, there were no bone fractures, a low risk of local recurrence (88% five-year local recurrence-free survival), low rates of radiation toxicities, and favorable functional outcomes.

Surgical margin has an important impact on outcome because it may be the only independent risk factor under the surgeon’s control in the treatment of an extremity STS [14, 40, 41]. Other well-known risk factors such as tumor size, grade, depth, and patient age are considered non-modifiable at disease presentation [14]. The definition of a “safe” surgical margin continues to evolve during the limb-salvage era. There are certain context-specific differences in terms of local oncologic control based on margin status [40, 41]. For superficial STS, or small and deep STS, surgery alone can provide a high degree of local control as long as true wide negative resection margins (i.e. 1–2 cm of surrounding normal tissue or a fascial barrier) can be obtained. Gerrand et al. classified positive margins into low and high-risk groups based on the risk of local recurrence [41]. They found that microscopic positive margins that occurred following planned dissections close to major blood vessels, motor nerves, or bone, in order to spare those critical structures, were associated with low rates of local recurrence when combined with radiation therapy. In contrast, an unplanned positive soft-tissue margin or a positive margin obtained following re-excision to salvage an unplanned excision performed elsewhere with positive margins, were both associated with local recurrence rates greater than 30%. Therefore it is best to avoid positive margins in either of these scenarios, if at all possible, in order to achieve a good outcome. In the case of a previously unplanned excision, although there is no association between the detection of sarcoma at the second procedure and the initial size or grade of the tumor, use of preoperative radiation, or the time lapse between interventions, identification of tumor in the re-excision specimen pathologically does significantly increase the risk for local tumor relapse [42]. At our institution we advise wide re-excision if possible for all patients who present following an initial unplanned excision with positive margins.

**Figure 3.**
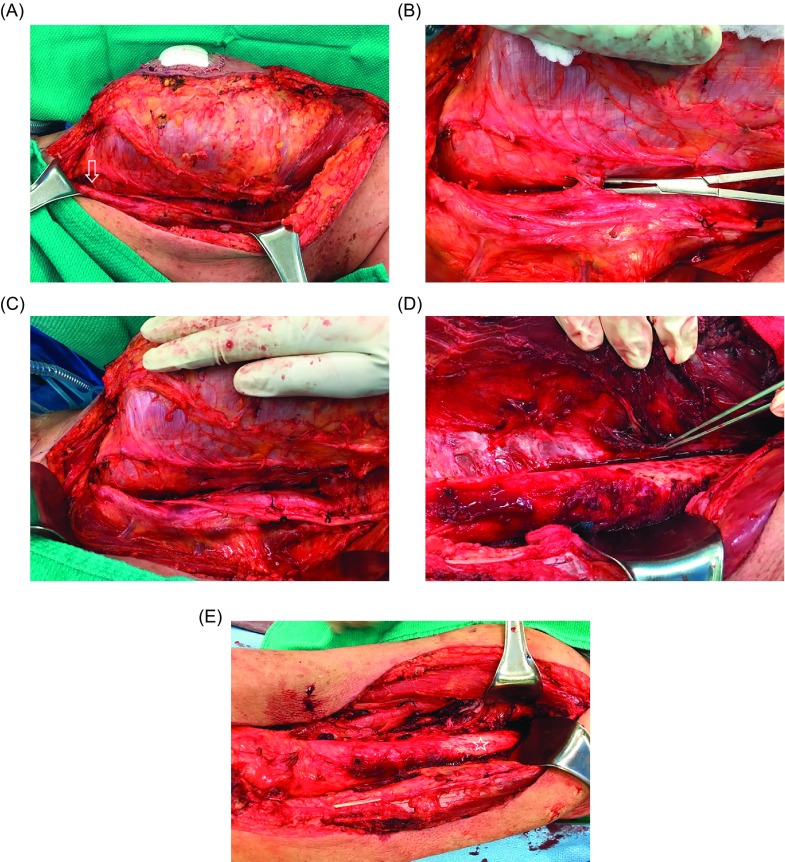
At the time of surgical excision (A), the femoral neurovascular bundle was very close to the tumor (arrow), with multiple perforating blood vessels entering the tumor (B). Due to preoperative IMRT it was safe to create a dissection plane between the tumor and the neurovascular bundle (C). The periosteum was also raised from the femur (pointer) as a margin along the tumor in the region where it was adherent to the bone (D). Although preoperative imaging showed the tumor to be very close to bone along the entire length of the femur, it was actually adherent to bone over a shorter length, so that only a small portion of the periosteum had to be removed (star) from the femoral shaft (E). The final pathological tumor resection margins were negative.

Other studies have assessed the safety and efficacy of close dissection along bone or critical neurovascular structures to facilitate limb salvage combined with radiation therapy (Figure 3). Clarkson et al. found no difference in local or systemic recurrence rates when epineural dissection was performed for buttock or thigh STS in order to preserve the sciatic nerve [43]. O’Donnell et al. showed that a positive margin following a close dissection to spare a major neurovascular structure or bone is relatively safe in terms of local recurrence, but is associated with worse cause-specific survival [44]. This study also showed that if a nerve or vessel is surrounded by tumor or a bone is invaded, complete resection of that structure en-bloc with the tumor to facilitate negative margins did not improve systemic disease control. Therefore the biology of each tumor plays a critical role in determining the ultimate oncologic outcome for the patient. These results suggest that critical structures can be preserved, in the context of multidisciplinary treatment, unless they are invaded or completely encased by tumor [43–46]. In cases where tumor invades into bone it may be necessary to resect a segment of bone and reconstruct the osseous defect in order to obtain an adequate surgical margin and facilitate limb salvage [10]. Otherwise a periosteal margin provides adequate local control when combined with adjuvant radiation (Figure 3).

Fractures in the radiation field can be particularly problematic because radiated bone does not reliably heal. Radiation-related fractures most commonly affect the femur and are significantly more frequent following postoperative radiation, likely due to the combination of higher radiation dose and larger treatment field [47–49]. High-risk patients can be identified using patient and treatment variables which have been combined into a nomogram (Table 2) [50], as well as specific bone radiation-avoidance principles [48]. Patients who are identified as high risk for developing a pathological fracture should have close follow-up and may benefit from prophylactic internal fixation, either at the time of the procedure or in a staged fashion [50].

**Table 2.** Risk factors for radiation-associated pathological femur fracture.

Risk factors [48, 50]
1) Increasing age at index procedure.
2) Large tumor size.
3) Location of tumor (anterior thigh compartment is at greatest risk).
4) Degree of periosteal stripping.
5) Female gender.
6) Postoperative radiation.
7) High dose radiation to bone based on bone avoidance principles.

## Soft-tissue reconstruction

The primary goal of oncologic surgery is to achieve negative surgical margins, and this often means that large soft-tissue defects are left following resection which are not amenable to primary wound closure [51]. Soft-tissue reconstruction following resection of a STS follows the theory of orthoplastic reconstruction based on the “reconstructive ladder” as described by Levin [52]. According to this protocol, the reconstructive surgeon uses the simplest procedure to cover a wound (e.g. primary closure) and then proceeds to more complex procedures to achieve wound closure and maximum functional benefit.

### Skin grafting

Following primary wound closure; skin grafting is the first rung of the reconstructive ladder. Split thickness skin grafting (STSG) can be used in a well-vascularized wound to cover muscle and tendons with paratenon. A historic contraindication to STSG was a radiated wound bed; however, recent studies have shown that a STSG can provide durable coverage of a radiated wound, and healing may be enhanced with a negative pressure wound dressing [53].

### Flaps

If the soft-tissue defect following STS resection precludes primary wound closure, and/or there are exposed nerves, vessels, bone, tendon without paratenon or hardware, the wound will need flap coverage as opposed to skin grafting. Flaps can be either local pedicled flaps or free flaps depending on the anatomic location and size of the defect requiring coverage (Table 3). Rotational flaps are highly predictable but recent advances have reduced the failure rate of free flaps to 1–4% [54]. Historically it was thought the vascular anastomosis for a free flap needed to be outside the zone of radiation; however, a recent study by Townley et al. showed that preoperative radiation does not increase the rate of microvascular complications [38].

**Table 3.** Common flaps for extremity reconstruction.

Type of flap	Free vs. Pedicled	Pedicle	Indication
Fasciocutaneous flaps			
Radial Forearm	Free or Pedicled	Radial artery antegrade or retrograde	Smaller soft tissue defects, exposed tendons, bone, joints, or neurovascular structures
Anterolateral thigh (ALT)	Free or Pedicled	Descending branch lateral femoral circumflex	Large soft-tissue defects, coverage of exposed tendons, bone, joints, and neurovascular structures
Muscle flaps			
Latissimus dorsi	Free or Pedicled	Thoracodorsal	Large soft-tissue defects with exposed bone, hardware, and neurovascular structures. Functional restoration of the elbow
Rectus abdominis (TRAM or VRAM)*	Free or Pedicled	Deep inferior epigastric	Large soft-tissue defects with exposed bone, hardware, and neurovascular structures
Gracilis	Free	Medial femoral circumflex artery	Medium soft-tissue defects with exposed bone, hardware, and neurovascular structures. Can also be innervated as a functional reconstruction
Gastrocnemius	Pedicled	Medial or lateral sural artery	Medium soft-tissue defects around the proximal tibia and knee. Functional restoration of the extensor mechanism of the knee

* TRAM = transverse rectus abdominis myocutaneous flap and VRAM = vertical rectus abdominis myocutaneous flap.

## Functional outcome

Following limb-salvage surgery or amputation, patients may be left with significant physical and emotional disability and reduced overall quality of life (QOL). In order to evaluate the impact of these procedures on patients, various functional assessments have been utilized. The most commonly used outcome measures are the Toronto Extremity Salvage Score (TESS) and the Musculoskeletal Tumor Society (MSTS) scoring system [55–57]. The TESS is a patient reported questionnaire which is validated to assess activity limitations, while the MSTS-87 is a physician rating based on function at specific anatomic locations (e.g. hip, knee) while the MSTS-93 is a physician rating based on function of the entire extremity (upper vs. lower) [55–57].

Resection of extremity STS is frequently a very invasive procedure, which can significantly impact a patient’s life; however, a majority of patients are left with moderate-to-high function following limb salvage [32, 58] (Table 4). Predictors of worse functional outcome following resection of STS include large tumor size, high-grade tumors, deep tumors, resection of bone, and sacrifice of a major motor nerve [58, 59]. In addition, patients’ inability to partake in life roles following treatment, [60] and their preoperative expectations [61] can have a significant impact on eventual QOL. Surprisingly additional factors, which may impart worse disease-specific survival and increase the risk of postoperative complications, may not impact the functional outcome. These factors include a radiation-induced sarcoma, [62] need for vascular reconstruction, [46] anatomic location, [59, 63, 64] and use of a free or pedicled flap [63, 65].

**Table 4.** Functional outcome following sarcoma resection.

Paper	Patient population	Comparison	Outcome measure	Impact on functional outcome
Davis et al. [58]	Lower extremity STS	Function of patients with limb salvage	MSTS 87 MSTS 93 TESS SF-36	Large tumor size: Lower extremity MSTS 1987, MSTS 1993, TESS Motor nerve resection (femoral, obturator, sciatic, peroneal, and posterior tibial nerves): Lower MSTS 1987, MSTS 1993, TESS Postoperative complications” Lower MSTS 1987 High-grade tumors: Lower MSTS 1993 and TESS Bone resection: Lower MSTS 1993
Davis et al. [32]	Extremity STS	Pre- vs. Postoperative radiotherapy	MSTS TESS SF-36	Postoperative radiotherapy: Improved MSTS, TESS, and SF-36 at 6 weeks postoperative only SF-36 compared to normative data: Lower for both treatment arms across all time points Wound complications: Lower MSTS at 6 weeks, 3, 6, 12, and 24 months Increased disability compared to baseline TESS Large tumor size (>10 cm): Lower MSTS scores at 6, 12, and 24 months Motor nerve resection: Lower MSTS scores Previous unplanned excision: Lower TESS score at 3, 6, 12, and 24 months
Davis et al. [31]	Extremity STS	Late morbidity: Pre- (50 Gy) vs. Postoperative (66 Gy) radiotherapy	MSTS TESS	Subcutaneous fibrosis: Decreased MSTS and TESS Joint stiffness: Decreased MSTS and TESS Extremity lymphedema: Decreased MSTS and TESS Pre- vs. Postoperative radiotherapy: No difference in MSTS or TESS Trend toward greater fibrosis with postoperative radiotherapy
Payne et al. [63]	Upper extremity STS with flap coverage	Pedicled vs. Free flap for wound coverage	MSTS 87 MSTS 93 TESS	Pedicled vs. free flaps: Decreased MSTS 87 from pre- to postoperative in patients with either pedicled or free flap Decreased MSTS 93 for free flaps No difference in TESS between groups Patients rated their function better compared to the actual rated impairment
Davis et al. [66]	Lower extremity limb salvage sarcoma patients	Relationship of symptoms to function during 1st year postoperative	Stiffness Fatigue Pain Weakness Limited range of motion TESS	Stiffness: Plateaus at 3 months Remains constant over the year Fatigue: Plateaus at 3 months Remains constant over the year Pain: Constant for 3 months then declines over study Weakness: Constant for 3 months then declines over study Limited Range of Motion: Constant decline over study TESS: Presence of pain, stiffness, weakness, and limited range of motion were predictors of worse outcome
	
Gerrand et al. [59]	Lower Extremity Limb Salvage Sarcoma patients	Sarcoma location and functional outcome: Groin/Femoral triangle Buttock Anterior thigh Medial thigh Posterior thigh Popliteal fossa Posterior calf Anterolateral leg Foot and ankle	MSTS 93 TESS	Deep vs. superficial: Superficial tumors have improved MSTS and TESS scores Superficial tumors: No decrease in MSTS or TESS from to pre- to postoperative Deep Tumors: No difference in MSTS or TESS based on tumor location Groin/Femoral triangle tumors: Increased pain based on the MSTS compared to other anatomic areas Decreased ability to sit, put on socks, getting in and out of bath, bending to pick up items More likely to have a limp or gait handicap Buttock/Posterior thigh: Decreased ability to sit
Ghert et al. [46]	Lower extremity limb salvage sarcoma patients	Vascular reconstruction and functional outcome: Femoral Iliofemoral Popliteal Tibial/Peroneal	TESS	Vascular reconstruction: More likely to need a muscle flap, have a wound complication, sustain a deep vein thrombosis (DVT), suffer from edema of the limb, and require an amputation No difference in the postoperative TESS
Jones et al. [45]	Lower extremity limb salvage sarcoma patients	Nerve resection and functional outcome: Femoral Sciatic Comparison of femoral nerve resection and location: Gender-matched large anterior thigh All large anterior thigh	MSTS 87 MSTS 93 TESS	Femoral nerve resection: No difference in MSTS 87, MSTS 93, or TESS between patients with sciatic nerve resection, gender-matched large anterior thigh tumors, or all patients with large anterior thigh tumors Long-term risk of falling which could lead to fracture
Pradhan et al. [67]	Patients with STS of the adductor compartment	Outcome of treatment of adductor compartment STS	TESS	Impact on TESS: Wound complications and high-grade tumors had lower TESS Timing of radiotherapy (pre- vs. postoperative) had no effect on TESS Need for a muscle flap; had no effect on TESS
Riad et al. [62]	Patients with radiation induced STS	Outcome of treatment in patients with a radiation-induced STS compared to patients with a sporadic STS	MSTS 87 TESS	Radiation induced vs. Sporadic STS: No difference in the MSTS 87 or TESS

MSTS 87 = Musculoskeletal Tumor Society Functional Rating System 1987, a measure of impairment; MSTS 93 = Musculoskeletal Tumor Society Functional Rating System 1993, a measure of impairment; TESS = Toronto Extremity Sarcoma Salvage Score, a measure of functional disability; SF-36 = 36-Item Short Form Health Survey, a quality of life measure.

## Surveillance

The majority of local recurrences as well as lung metastases will become evident within the first two years following treatment. As a result, high-risk patients are seen in follow-up every three months for the first two years for clinical examination and a chest x-ray or CT-scan. We only perform MRI of the surgical site or lymph nodes as part of regular follow-up if there is clinical concern for local or regional recurrence, based on physical examination in the clinic or changes noted by the patient. After the first two years high-risk patients are reviewed every six months until five years and then annually until 10 years.

## Summary

Extremity STS are aggressive and rare malignant tumors with several factors such as size, depth, grade, and tumor location which influence outcome. Following a tissue diagnosis and staging, the treatment of patients with STS involves a multidisciplinary team approach and most patients are eligible for limb-salvage surgery, usually combined with radiation. Following treatment the majority of patients can expect a painless and functional extremity.

## Conflict of interest

No conflicts of interest are declared by any author on this study.
